# Analysis of the healthcare waste management status in Tehran hospitals

**DOI:** 10.1186/s40201-014-0116-4

**Published:** 2014-08-27

**Authors:** Fariba Malekahmadi, Masud Yunesian, Kamyar yaghmaeian, Kazem nadafi

**Affiliations:** Environmental and occupational Health centre, Ministry of Health and Medical Education, 11th floor, Simaye Iran St, Tehran, 1467664961 Iran; Department of Environmental Health Engineering, School of Public Health and Institute for Environmental Health Engineering Tehran University Of Medical Sciences, Tehran, Iran

**Keywords:** Healthcare waste, Healthcare waste management, Segregation, Collection, Transportation, Temporary storage, Treatment, Disposal

## Abstract

**Background:**

Considering the importance of healthcare waste management, following the ratification of the Waste Management law in 2005 and the subsequent approval of its executive bylaw in 2006 and finally the healthcare waste management criteria passing by the parliament in 2008, a review on the status of healthcare waste management is needed to implement the mentioned law properly.

**Findings:**

In this retrospective study during six months period all public hospitals in Iran’s capital city, Tehran, were selected to conduct the survey. Data collected through an expert-standardized questionnaire was analyzed by using SPSS software. The results of the current status of healthcare waste management in Tehran hospitals showed 5.6% of hospitals were ranked excellent, 50.7% good, 26.4% medium, and the 13.9% of hospitals were ranked weak and 3.5% ranked very poor.

**Conclusions:**

The findings showed that appropriate technologies should be used to have better disposal stage. As the ratified criteria were not fully observed by all the selected hospitals, training courses and comprehensive program conducting by each hospital could be enjoyed as practical tools to implement the all stages of healthcare waste management properly.

## Introduction

Over the past few years, there has been an increase in the public concern about the management of healthcare wastes all over the world [1]. An estimate shows that 5.2 million people (including 4 million children) die from waste-related diseases per year [2]. Healthcare waste includes all the waste generated by healthcare establishments, research facilities, and laboratories. The type of generated wastes depend on numerous factors such as established waste management methods, type of healthcare institutions, hospital specializations, proportion of reusable items employed in healthcare centers, and number of patients treated daily [3]. Of the total amount of waste generated by healthcare activities, about 80% is general waste. The remaining 20% is considered hazardous material that may be infectious, toxic or radioactive [4]. Although infectious waste is only a small part of the total waste generated by medical facilities, it accounts for a considerable portion of the costs incurred by a healthcare facility for the disposal of medical waste [5]. Healthcare waste specifically has a high potential of carrying microorganisms that can infect people exposed to it, as well as the community if it is not properly disposed [6]. Therefore, when hazardous healthcare wastes are not properly managed, exposure to them could lead to infections, infertility, genital deformities, hormonally triggered cancers, mutagenicity, dermatitis, asthma and neurological disorders in children; typhoid, cholera, hepatitis, AIDS and other viral infections through sharps contaminated with blood [7]. Epidemiological studies indicate that a person who experiences one needle-stick injury from a needle used on an infected source, patient has risks of 30%, 1.8%, and 0.3% respectively to become infected with HBV, HCV and HIV [8]. Healthcare waste management includes all activities involved in waste generation, segregation, transportation, storage, treatment and final disposal of all types of waste generated in the healthcare facilities, stages of which require special attention [9].

Mismanagement of hospital waste implies a combination of improper handling of waste during generation, collection, storage, transportation and treatment [10]. Healthcare waste is poorly managed at too many health care facilities worldwide. Identifying the causes and then supporting improvements in the system are key skills that healthcare facility managers need to develop [11]. Urban and rural hospitals and clinics in developing countries dispose their medical waste in a manner that pose a risk of disease among populations. In 2002, the results of World Health Organization (WHO) assessment conducted in 22 developing countries showed that the proportion of healthcare facilities that did not use proper waste management was significant, ranging from 18% to 64% [12]. The spread of blood-borne pathogens in health-care waste motivated the WHO to issue a policy in 2004 calling for the development of national policies, guidance, and plans for healthcare waste management [13]. All approaches to the management of healthcare waste must consider the environmental, financial, and technical feasibility of treatment and disposal technologies (Program for Appropriate Technology in Health [14]. Following the mentioned descriptions, the great attention has been paid to healthcare waste issue in Islamic Republic of Iran, which is lead to the ratification of the “waste management Low” in 2005, and the subsequent approval of its executive bylaw in 2006. According to 11th article of this low, rules and methods of executive healthcare waste management should been ratified by cooperation of environmental organization and health ministry. Following that, the criteria based on WHO and Environmental Protection Agency (EPA) guidelines were ratified by Islamic parliament in 2008. These criteria involved 8 chapters and 4 appendixes contained issues about medical waste segregation, collection, storage, transportation, treatment, and disposal. Table of medical waste classification, treatment and disposal methods criteria, the characteristics of medical waste bags, and personal safety devices recommended for workers responsible for waste transportation are issues in the appendixes. Based on 7th article of waste management law, waste producer is responsible for executive management of medical waste. Therefore, hospitals should implement an appropriate way to manage their wastes according to the ratified executive criteria. In order to proper programming and adequate implementation of ratified criteria, this research was conducted in which by analyzing the current status of medical waste management, the problems were find and the practical recommendations were given.

## Findings

144 hospitals in city of Tehran have been investigated in order to collect the general and specific information to indicate the current status of healthcare waste management. A questionnaire contained 72 questions addressing the main phases of the waste management process in 6 parts including segregation, collection, transportation, storage, disposal, as well as the hygiene status separately, were used to collect the data.

To convert the data into a quantitative measures, score 1 was assigned to answers which were complied with the law and score 0 was assigned to those which were not complied with the law. Then in each separate part of the questionnaire, indicating each phase of healthcare waste management, scores were converted in to 0–100 to be ranked as shown in Table 1. Subsequently, the mentioned ranked scores were scaled up in the expert group as shown in Table 2. The mentioned scaled up scores are used as the basis of descriptive statistical analysis.

**Table 1:** **The ranges for ranking the hospital waste management**

**Range**	**Rank**
91-100	Excellent
71-90	Good
51-70	Medium
26-50	Poor
0-25	Very poor

**Table 2:** **The weighting factors for healthcare waste management status**

**Healthcare Waste management process**	**weight**
Segregation	20
Collection	20
Transportation	25
Temporary Storage	10
Treatment and disposal	25

The frequency tables and the central and dispersion index of descriptive statistics were used in order to analyze the available data including mean, median, and standard deviation. To indicate the normal description of healthcare waste management process, the non parametric test (1-sample k-s) was used. Considering the fact that p < 0.05, Kruskal–Wallis and Mann–Whitney tests were used to investigate that whether or not these parameters (hospital ownership, hospital ranking, hospital activity, involving environmental expert in hospital, and holding healthcare waste management training course in the hospitals) influence healthcare waste management.

The average waste generation in hospitals was 2.9 kg/bed per day. The density of healthcare waste rates ranged from 120 to 150 kg/m3, and the total average of healthcare waste generation was 65000 kg/day. It should be mentioned that pathological wastes were segregated and collected considering ratified regulations of Iran Health Ministry included healthcare wastes management issues.

Most of the selected hospitals (84.7%) have environmental health expert responsible for hospital’s healthcare waste management. But, there are few hospitals (15.3%) that have no experts to monitor the healthcare waste management status in the hospital.

Training course considering healthcare waste management was held in 52.78% of the hospitals, but 47.22% of the hospitals had no training courses on this issue.

Though the ratified low emphasis on having executive program for healthcare waste management, 75.69% of hospitals did not have such a program.

Considering segregation status, 51.4% were ranked medium, 27.1% were ranked good, and 10.4%, 9%, 2.1% were ranked poor, excellent, and very poor respectively.

Most of the hospitals were ranked excellent in collection stage (43.1%). In contrast, the study in India shows the worst collection status for healthcare wastes as wastes were collected in a mixed form, transported and then disposed of along with municipal solid wastes [15].

Most of the hospitals (39.6%) were in excellent status considering transportation process. 29.9%, 14.6%, 4.9%, and 11.1% were ranked good, medium, poor, and very poor respectively. The study investigated in Irbid city (a major city in the northern part of Jordan) showed that healthcare facilities in Irbid city have less appropriate practices when it comes to the handling, storage, and disposal of wastes generated in comparison to the developed world. There are no defined methods for handling and disposal of these wastes, starting from the personnel responsible for collection through those who transport the wastes to the disposal site. Moreover, there are no specific regulations or guidelines for segregation or classification of these wastes [16].

In the storage stage, 43.1% of hospitals were ranked excellent, 34% of hospitals good, 13.2% of hospitals medium, 2.1% of hospitals poor, and 7.6% of hospitals were ranked very poor. The research in UK showed that storage of clinical waste carts in areas accessible to members of the public and failure to lock individual waste carts was common. Waste segregation was poor. Many clinical waste carts and the areas dedicated to their storage were in a poor state of repair [17].

Most of the hospitals were in poor status considering disposal process. 24.3% of hospitals were in very poor situation. 8.3%, 5.6%, and 4.9% were ranked as excellent, good, and medium respectively. The waste disposal has the lowest average in comparison to the other process of healthcare waste management in this study due to not using appropriate technologies. Therefore, the hazard of healthcare waste could impacts the public negatively, though all efforts in the other healthcare waste management stages were satisfactory. The study in Egypt revealed that, the current situation of medical waste disposal in Alexandria is depending on incinerators. Some of these incinerators are not working anymore. Incinerations as a system is not accepted at the time being in most developed countries such as Iran due to the risks associated with it and suitable substitution management system for healthcare waste disposal is now taking its place [18].

The Kruskal –Wallis Test considering P < 0.05 and The Mann–Whitney Test considering P < 0.05 showed that there was a correlation between waste management different process and hospital rank. As the hospitals ranked excellent with 75.77 median are in better condition in healthcare waste management in comparison with other hospitals ranked as good, medium, poor, and very poor with 68.61, 62, 58.3, and 45.14 median respectively.

There was also a correlation between healthcare waste management and having environmental health expert. (p = 0.017). The waste management status in hospitals having such experts was in the better situation with 68.68 mean in comparison with other hospitals without mentioned experts with 58.58 mean. It should be mentioned that the waste management status in 53.3% of hospitals having environmental experts ranked good.

There was a correlation between healthcare waste management status and holding training course involved healthcare waste issues considering P < 0.001. The healthcare waste management was good in 69.1% of hospitals with trained staff.

**Figure 1:**
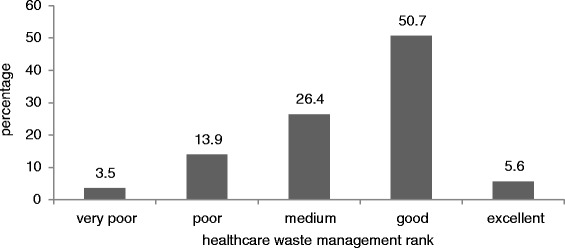
**Percentage of each rank for waste management.**

The percentage of healthcare waste management ranks is shown in Figure 1. It can be seen that most of the hospitals were ranked good. The comparison between Tehran hospitals with Karachi hospitals showed that Tehran hospitals were in more appropriate situation. Out of eight hospitals visited 2 (25%) were segregating sharps, pathological waste, chemical, infectious, pharmaceutical and pressurized containers at source. For handling potentially dangerous waste, two (25%) hospitals provided essential protective gears to its waste handlers. Only one (12.5%) hospital arranged training sessions for its waste handling staff regularly. Five (62.5%) hospitals had storage areas but mostly it was not protected from access of scavengers. Five (62.5%) hospitals disposed off their hazardous waste by burning in incinerators, two (25%) disposed of by municipal landfills and one (12.5%) was burning waste in open air without any specific treatment. No record of waste was generally maintained. Only two (25%) hospitals had well documented guidelines for waste management and a proper waste management team [19].

The study conducted in 15 cities in Nanjing (a city in China) showed that the healthcare waste management in this city is rather similar to the mentioned management in Tehran city. As a case in point, the segregated collection of various types of healthcare waste has been conducted in 73% of hospitals. Furthermore, 93.3% of the hospitals have temporary storage areas [20].

The study conducted by Patil and Shekdar (2001) demonstrated that at many places, authorities are failing to install appropriate systems due to a variety of reasons, such as non-availability of appropriate technologies, inadequate financial resources and absence of professional training on waste management, while in Tehran at least in 27% of hospitals infectious wastes and sharps are disposed by using appropriate technologies.

## Conclusion

It can be concluded that the status of collection, temporary storage, and transportation were better than segregation and disposal process. Additionally, among the mentioned process the status of disposal is the most problematic. So, appropriate technologies should be used and the comprehensive program should be initiated to prevent adverse impacts of inappropriate disposal on the environment. As it was found that having environmental health experts besides holding training course in a matter of appropriate healthcare waste management influence healthcare waste management, these should be accepted in all hospitals. Considering the findings of this study, there are hospitals not obeying the ratified regulations. Thus, more comprehensive program should be conducted and practiced in hospitals to implement the mentioned regulations completely.
